# A Case of an Unusual Bleeder: Small Bowel Lymphoma

**DOI:** 10.7759/cureus.59448

**Published:** 2024-05-01

**Authors:** Eli A Zaher, Mohamed A Ebrahim, Parth Patel, Daria Zaher, Maen Talib

**Affiliations:** 1 Internal Medicine, Ascension Saint Joseph Hospital, Chicago, USA; 2 Internal Medicine, University Clinical Hospital in Bialystok, Bialystok, POL; 3 Internal Medicine, Midwestern University Chicago College of Osteopathic Medicine, Chicago, USA

**Keywords:** small intestinal lymphomas, gastrointestinal bleeding, ulcerative colitis, diffuse large b-cell lymphoma, b cell lymphoma

## Abstract

This case report highlights an uncommon presentation of small bowel lymphoma as gastrointestinal bleeding in an 87-year-old female with a history of ulcerative colitis. Despite non-specific symptoms and negative findings on upper endoscopy and colonoscopy, ileoscopy revealed a distal ileal mass with a solitary non-bleeding ulcer, confirmed by biopsy as diffuse large B-cell lymphoma (DLBCL). The patient opted for palliative management. Small intestinal lymphomas, particularly DLBCL, pose diagnostic challenges due to their varied presentations. Timely detection is crucial for optimal outcomes, emphasizing the importance of prompt utilization of diagnostic methods in suspected cases.

## Introduction

Gastrointestinal lymphoma is the most encountered extra-nodal lymphoma, although it is fairly uncommon [1]. Neoplasms of the small bowel constitute under 2% of all gastrointestinal malignancies and are predominantly found in the ileum [2,3]. Symptoms are generally non-specific, with bleeding being an extremely uncommon presentation [1]. We present the case of small bowel lymphoma presenting as a gastrointestinal bleed.

## Case presentation

An 87-year-old female with a history of ulcerative colitis status post-proctocolectomy with end ileostomy was brought to the emergency room from her nursing home after noticing multiple blood clots in her ostomy bag. She denied any associated dizziness, fevers, abdominal pain, bloating, reduced appetite, nausea, or vomiting. She likewise denied using any anticoagulants or non-steroidal anti-inflammatory drugs (NSAIDs). 

Her vital signs on admission were within normal limits. A physical examination revealed minimal bloody output in the ostomy bag. Her abdomen was otherwise soft and non-tender. Blood work-up was consistent with iron deficiency anemia without coagulopathy (Table 1).

**Table 1:** Blood work on admission

Component	Result	Reference range
Hemoglobin (g/dL)	7.8	12.0-15.3
White cell count (k/mm cu)	6.2	4.0-11.0
Mean corpuscular volume (f/L)	73	80.0-100.0
Platelets (k/mm cu)	211	150-450
Creatinine (mg/dL)	0.9	0.6-1.2
Ferritin (ng/mL)	5	12-150
International normalized ratio	1.0	0.9-1.1
Partial thromboplastin time (seconds)	25	21-35

**Figure 1:**
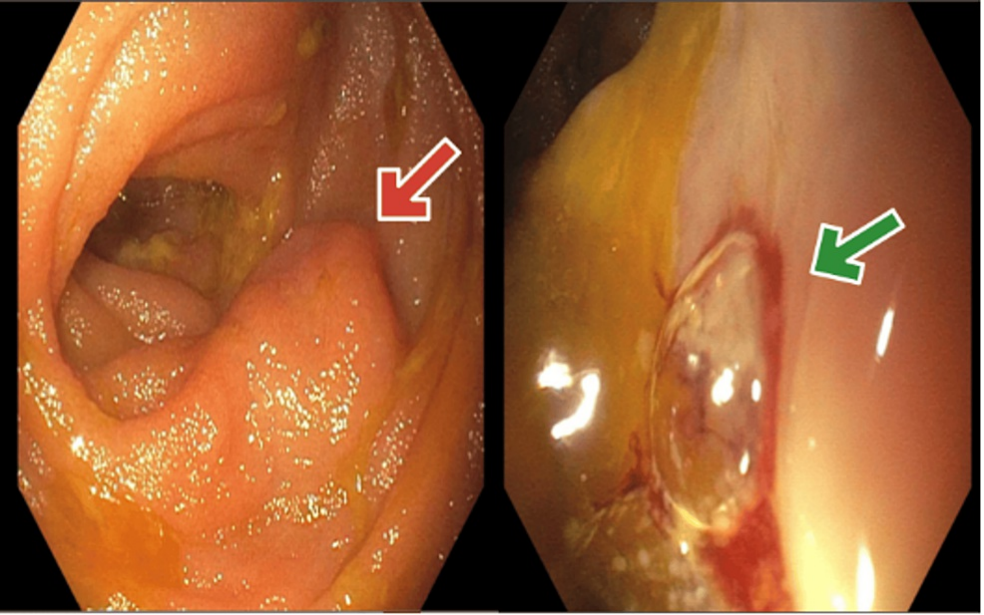
Findings on ileoscopy The red arrow points at a mass in the distal ileum. The green arrow points towards the bleeding site on the mass.

**Figure 2:**
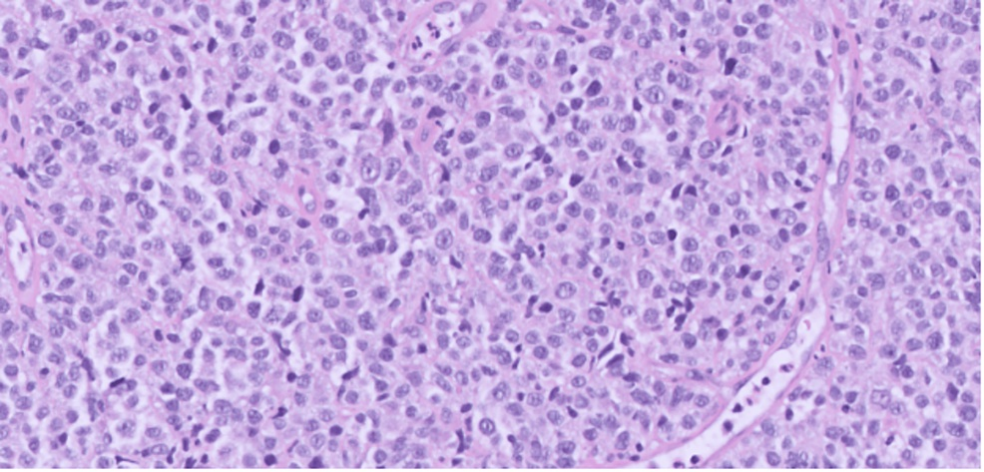
Histology of distal ileal mass

Upper endoscopy and colonoscopy failed to reveal the source of bleeding. The patient continued to have bloody output with a continuous drop in her hemoglobin, requiring a total of 2 units of blood transfusion. Therefore, an ileoscopy was performed and revealed a distal ileal mass with a solitary, non-bleeding ulcer (Figure 1). The biopsy of the mass confirmed DLBCL (Figure 2). The patient opted for palliative management and was discharged home with hospice care.

## Discussion

Neoplasms of the small bowel make up a small fraction, less than 2%, of all gastrointestinal cancers, with a significant majority, approximately 75%, being malignant. Among these malignancies, lymphoproliferative disorders constitute 15% to 20% of cases. The ileum is the most commonly affected site, accounting for 60% to 65% of small bowel neoplasms, followed by the jejunum (20% to 25%) and the duodenum (6% to 8%) [4]. Non-Hodgkin lymphoma (NHL) represents a heterogeneous group of malignancies capable of manifesting in diverse anatomical locations throughout the body, commonly affecting both nodal and extranodal structures within the abdomen. Nodal disease may present as a single mass or, more frequently, multiple masses. Solitary nodal lymphomas can appear as either a single, enlarged lymph node or the merging of several enlarged nodes [5]. Extra-nodal lymphomas, which originate from solid organs, constitute approximately one-third of NHL instances. These extranodal manifestations typically occur in areas such as the gastrointestinal tract, Waldeyer’s ring, skin, and bone. Within the gastrointestinal tract, the predominant histological subtypes of lymphoma are mucosa-associated lymphoid tissue (MALT) and diffuse large B-cell lymphoma (DLBCL), primarily of B-cell origin [4].

Symptoms of small intestinal lymphoma are varied and nonspecific, including abdominal pain, ileus, diarrhea, weight loss, gastrointestinal bleeding, palpable mass, and nausea [6]. In this instance, the patient exhibited symptoms of anemia and observed blood clots in her ostomy bag following a proctocolectomy with end ileostomy procedure undertaken to manage her ulcerative colitis condition, with investigations such as upper gastrointestinal endoscopy and colonoscopy showing no evident abnormalities. Given the nonspecific nature of signs and symptoms, the diagnosis necessitates the pathological assessment of adequate tissue samples. Lymphoma affecting the small intestine may present as a mass, polyp, or ulcer on capsule endoscopy, exhibiting features that cannot be differentiated from other lesions. Following the ileoscopy, it was observed that there was a mass in the distal ileum along with a solitary ulcer that was not actively bleeding. A biopsy of the terminal ileum confirmed the diagnosis of DLBCL.

Aggressive B-cell lymphoma presents a potential for cure, with treatment strategies tailored to the specific pathology of the lymphoma. For extra-nodal B-cell lymphomas, the cyclophosphamide, doxorubicin, vincristine, and prednisolone (CHOP) regimen remains the primary chemotherapy approach. The integration of rituximab into chemotherapy has notably enhanced the prognosis of DLBCL patients, enhancing complete response rates and improving event-free and progression-free survival rates [7]. With the advent of rituximab, the necessity for surgery has somewhat diminished. Nonetheless, findings from the Korean Lymphoma Study Group demonstrate superior three-year overall survival rates and lower relapse rates in intestinal DLBCL patients treated with both surgery and chemotherapy compared to chemotherapy alone (91% vs. 62% overall survival, 15.3% vs. 36.8% relapse rate, p < 0.001). Particularly in localized intestinal DLBCL cases, the combination of surgical resection and chemotherapy offers significant benefits with an acceptable tradeoff in terms of quality of life [8]. In our case, the patient opted for palliative management.

## Conclusions

Diffuse large B-cell lymphoma stands as the predominant form of primary extranodal lymphoma affecting the gastrointestinal tract. Patients diagnosed with primary small intestinal lymphoma often present with nonspecific symptoms, complicating both diagnosis and timely treatment initiation. In our case, the presenting symptom was gastrointestinal bleeding, which is a rare phenomenon. A delayed diagnosis can negatively impact patients' performance status. Therefore, it is crucial to promptly utilize accessible diagnostic methods to detect DLBCL in its early stages.
